# Experimental measurement and numerical modeling of deformation behavior of breast cancer cells passing through constricted microfluidic channels

**DOI:** 10.1038/s41378-023-00644-7

**Published:** 2024-01-12

**Authors:** Pouyan Keshavarz Motamed, Hesam Abouali, Mahla Poudineh, Nima Maftoon

**Affiliations:** 1 Department of Systems Design Engineering, University of Waterloo, Waterloo, ON N2L 3G1 Canada; 2 Center for Bioengineering and Biotechnology, University of Waterloo, Waterloo, ON N2L 3G1 Canada; 3 Department of Electrical and Computer Engineering, University of Waterloo, Waterloo, ON N2L 3G1 Canada

**Keywords:** Engineering, Physics

## Abstract

During the multistep process of metastasis, cancer cells encounter various mechanical forces which make them deform drastically. Developing accurate in-silico models, capable of simulating the interactions between the mechanical forces and highly deformable cancer cells, can pave the way for the development of novel diagnostic and predictive methods for metastatic progression. Spring-network models of cancer cell, empowered by our recently proposed identification approach, promises a versatile numerical tool for developing experimentally validated models that can simulate complex interactions at cellular scale. Using this numerical tool, we presented spring-network models of breast cancer cells that can accurately replicate the experimental data of deformation behavior of the cells flowing in a fluidic domain and passing narrow constrictions comparable to microcapillary. First, using high-speed imaging, we experimentally studied the deformability of breast cancer cell lines with varying metastatic potential (MCF-7 (less invasive), SKBR-3 (medium-high invasive), and MDA-MB-231 (highly invasive)) in terms of their entry time to a constricted microfluidic channel. We observed that MDA-MB-231, that has the highest metastatic potential, is the most deformable cell among the three. Then, by focusing on this cell line, experimental measurements were expanded to two more constricted microchannel dimensions. The experimental deformability data in three constricted microchannel sizes for various cell sizes, enabled accurate identification of the unknown parameters of the spring-network model of the breast cancer cell line (MDA-MB-231). Our results show that the identified parameters depend on the cell size, suggesting the need for a systematic procedure for identifying the size-dependent parameters of spring-network models of cells. As the numerical results show, the presented cell models can simulate the entry process of the cell into constricted channels with very good agreements with the measured experimental data.

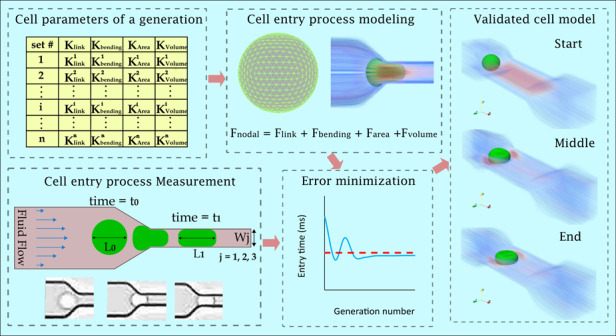

## Introduction

In addition to several genetic and biochemical factors contributing to the metastatic progression, the biomechanical forces are widely reported to be of considerable importance^1,2^. Among these forces, the hemodynamic forces play a critical role in metastasis progression, for the blood is the primary route of transporting the circulating tumor cells (CTCs) throughout the body^3,4^. Besides, CTC’s survival, intravascular arrest, and extravasation are significantly impacted by shear stresses exerted on them originating from their synchronous interactions with the blood plasma, blood cells, and endothelial cells that form the inner layer of blood vessels^1,5–7^. Despite the crucial importance of the impact of such interactions in the hematogenous spread of metastasis, our understanding is far from complete due to the complicated nature of the underlying phenomena. Advanced numerical methods, capable of accounting for the high deformability attribute of CTCs, cell-cell interactions, and interactions with the fluid flow in the delicate microcapillaries have been shown to be promising to decipher CTC’s responses to the exerted mechanical forces^8–11^. Priceless knowledge could be acquired by numerically investigating the impact of various sources of mechanical forces on CTCs. Such knowledge helps answer the perplexing question of when, where, and how a secondary tumor can be formed in a distant organ from migrated CTCs^5,12–14^. Furthermore, it enables the development of more accurate diagnostic methods for localizing secondary tumors and the discovery of more efficient treatments for preventing or delaying this deadly disease^12^.

However, for being trustable, a numerical model should numerically replicate the experimentally observed behavior of CTCs^15^. The deformation behavior of CTCs, that greatly affects every step of the metastatic cascade, has been documented to be one of the key characteristics of malignant CTCs in many research studies^1,16–18^. Thus, the deformation behavior of CTCs can be considered as one of the essential behaviors of CTCs that the numerical model must simulate accurately in comparison with the measured experimental data. Besides, numerical modeling of more complex phenomena such as cell-cell interactions, cell adhesion to vessel walls, and extravasation will be affected by the accurate representation of the deformation behavior of CTCs^9,19,20^. This necessitates developing an experimentally validated numerical model of single CTCs’ deformation behavior before moving toward modeling more complex phenomena. This requires acquiring precise measurements of single CTC’s deformability in a context similar to their microenvironment.

In vivo models that capture the motion and deformation of CTCs in real time are the most relevant models in terms of replicating the microenvironment^5,21^. Although, challenges with in vivo models such as capturing high-resolution images and having precise control of the fluid flow make in vitro models good alternatives for investigating the deformability of cancer cells^12,22^. Since microfluidic devices are the most relevant models in terms of similarity to the blood capillaries among in vitro models, developing advanced microfluidic devices to quantify migratory and deformability capabilities of human cancer cells has attracted many researchers^23–29^. In this regard, shear flow deformability cytometry^30,31^ and extensional flow deformability cytometry^32^ are the two contact-free approaches that utilize hydrodynamic flow to change the shape of the cells and assess the cell deformability from cell images captured during cell shape change period^33^. Although these contactless cytometry methods can evaluate cell deformability in a high throughput manner, they are not able to investigate the role of cell-wall interactions on cell deformability. In addition, constricted microfluidic channels whose width are comparable to microcapillary diameters (≈10 μm)^34^ became one of the most relevant tools to study the deformability of cancer cells. Using such devices has demonstrated that the entry time, which is the time it takes for the cell to squeeze and fully enter the constriction, would be impacted by the deformability of the cell and is sensitive enough to be different in cancer cells with different levels of malignancy^16,17,28,35^. In addition, since the constricted channel geometry, fluid flow rate, and cells motion are controlled accurately in this type of experiment, it is a good candidate that based on the numerical models of CTC’s deformability can be developed and validated. Furthermore, the numerical model is valid when cells are in close contact with the walls of the fluidic domain which could be the case for many practical biomedical applications^21^. More specifically, the cells’ entry times calculated from the numerical model can be validated against the experimentally measured ones^8,36–38^.

To date, various numerical methods have been established to quantitatively model the entry process of deformable objects into narrow confinements^37,39–46^. Most precedent models are continuum models which span from liquid drop models to solid models^47^ that considered a cell as a viscous fluid encapsulated with the cell’s membrane which obeys related constitutive equations^37,39–41^. However, solid models suffer from accurately modeling large deformation of cells, and liquid drop models are insufficient in simulating cell-wall interactions since they assume a lubrication layer between the cell and the constriction walls^41,47–49^. Although solid continuum models with hyperelastic constitutive laws can improve the accuracy of results for large elastic deformation of cells^50,51^, in general, the applicability of continuum cell models for modeling complex biological phenomena have been limited due to their computational inefficiency in dealing with multiple deformable bodies and integration of biochemical interactions at the cellular scale^52^.

To obviate the mentioned drawbacks, spring network models (SNM) of the cell membrane have been devised, progressed, and applied^53–58^. SNM, discretized the cell membrane by a triangular spring network, was devised based on the idea of imitating the physics of the cell membrane by using springs in the numerical model instead of spectrin on the membrane of a red blood cell (RBC)^53,54,59^. High computational efficiency without dropping the model accuracy has been reached with the more advanced SNMs by decreasing the vertices on the cell membrane^55,60,61^. This makes such a method practical for scaling up for simulating circulation, entrapment, and extravasation of CTCs for vessels and organ scale models of metastasis. Moreover, the computational efficiency and accuracy of SNM over continuum models of RBCs suspended in the fluid was demonestrated^49^. More specifically, a comparison between the computational performance of a spring network model and a continuum linear elastic solid model^62^ of RBC proved the higher computational efficiency and higher accuracy of the spring network model over the continuum linear solid model.

However, SNMs have a few unknown model parameters, whose quantities affect the deformation behavior of cells directly, need to be identified for each cell type, cell size, and mesh arrangement to replicate the correspondence experiment accurately. The first efforts of finding these parameters were performed on RBC by either manual adjustment or by making a link between SNM and continuum models^57,63,64^. Since these efforts suffered from unrealistic assumptions in identifying the parameters of RBC, the results were not accurate in replicating the experimental data and other rounds of calibration were required^56,64^. In addition, being highly uncertain in finding the unknown parameters of SNM hinders applying this model to other cell types such as CTCs. Recently, a systematic approach called SNM-GA for identifying SNM parameters has been developed to minimize the error between the experimental data and computational calculation using a genetic algorithm (GA) running on supercomputers^8^. SNM-GA is an inverse method that requires appropriate experimental data that should make the identification step feasible and assure producing a validated numerical model able to simulate the deformation behavior of CTCs robustly. Considering the cells’ entry process, the data must be reported as cell’s entry time and the quantity must be in the order of milliseconds to help SNM-GA identify the parameters feasibly without needing huge computational resources. Besides, measuring the entry time in various devices with various constriction widths increase the number of inputs and outputs that will be used in the parameter identification step and is the key for developing a robust numerical model^8^. To the best of our knowledge the literature lacks such experimental data that reported the entry time of breast cancer cells in order of millisecond using various micro constriction widths. Therefore, capturing such experimental data was one of the goals of this study.

In this study, we first experimentally investigated the deformability of three different breast cancer cell lines (MCF-7, SK-BR-3, and MDA-MB-231) using a constricted microfluidic device whose constriction width is 10 μm. Then, by focusing on the more deformable one (MDA-MB-231), we extended the experiments to two additional constricted microfluidic devices with 8 μm, and 12 μm widths of constriction. Using SNM, the numerical models of a single cancer cell passing through constricted channels have been developed. Afterward, the unknown parameters have been identified based on the measured entry times with the use of GA by minimizing the error between computational calculations and the experimental measurements. We demonstrated that by applying the identified model parameters, both the gradual squeezing of the cell into the constricted channels and the shape change of the cell during the entry process could be accurately replicated in-silico. This study is the first study to present a valid discrete numerical model for the deformation behavior of highly metastatic cancer cells (MDA-MB-231) for a range of cell diameters. The presented models can be used as the foundation of discrete models of the motion and deformation of circulating breast cancer tumor cells upon which numerical models of more complex phenomena such as cell-cell interactions, and CTC cluster deformation behavior in microcapillaries can be built.

## Results and discussion

### Experimental measurement

**Fig. 1:**
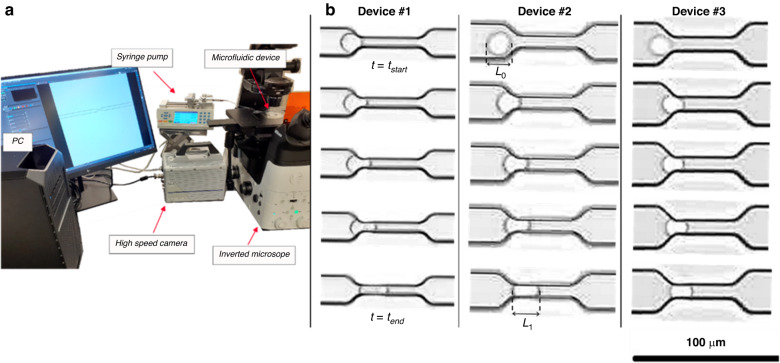
Experimental set up for cancer cell deformability measurement. **a** Experimental set up for high-speed measurements of the single breast cancer cell deformability. **b** Captured images from entry process of the single breast cancer cell with the diameter of 18.2 µm (passing device #1), 22.3 µm (passing device #2) and 17.9 µm (passing device #3) at five instances using the three constricted microfluidic devices (Supplementary information Video 1) Entry time starts when cell starts entering the constriction and ends when it fully enters the constriction $({{\bf{Entry}}\; {\bf{time}}}={\boldsymbol{t}}_{{{\bf{end}}}}-{\boldsymbol{t}}_{{\bf{start}}})$. Elongation index is the ratio of the cell length after complete entry to the undeformed cell length at the start of the entry $({{\bf{Elongation}}\; {\bf{index}}}=\frac{\boldsymbol{L}_{1}}{\boldsymbol{L}_{0}})$

In this study, to investigate the deformation behavior of breast cancer cells in a microcapillary similar to that of CTCs passing confined spaces, we measured the entry process of the cells entering narrow constricted microfluidic channels. Samples of captured images of the entry process of cancer cells (MDA-MB-231) in all three microfluidic devices (Device #1 to Device #3 from left to right) at five instances are shown in Fig. 1. As shown in Fig. 1b, the entry time starts when the cell starts entering the constriction and ends when the cell fully enters the constriction. In addition, the elongation index, is defined as the ratio of the cell length after completing the entry to the constriction to the original length of the cell before the start of the entry process as shown in Fig. 1b. The images were selected to show similar location and deformation of the cells, but the timing of the squeezing process is different in each device as detailed below.

**Fig. 2:**
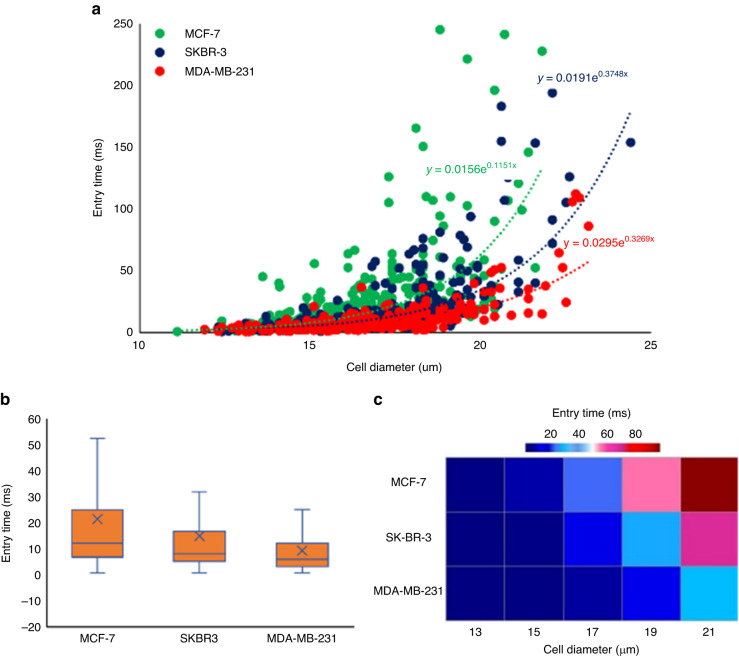
Deformability measurement of breast cancer cell lines with various level of invasiveness. Comparison of the deformability of breast cancer cell lines (MCF-7, SK-BR-3, and MDA-MB-231) using device #2 by illustrating the measured data with **a** scatter graph showing all points of entry time vs cell diameter for every single cell, **b** box plot of entry time of all captured data in the three cell lines, and **c** the heat map for average entry time in various cell diameter

First, we compared the cell deformation behavior of different breast cancer cell lines MCF-7, SK-BR-3, and MDA-MB-231 with moderate to high metastatic potential^65^ (Fig. 2). In Fig. 2a, each point represents one measured cell. As measured data in Fig. 2a show the entry times of all cell lines are increasing exponentially

as the cell diameter increases. A comparison between the exponential rate of the best-fit curves of all three cell lines discloses that the entry time of MDA-MB-231 is less sensitive to the increase of cell diameter than the other two cell lines. In addition, entry times of highly invasive breast cancer cells (MDA-MB-231) are shorter than the other two cell lines for a given cell size as the exponential fits in Fig. 2a shows. Taking the cell diameter of 18 μm, the entry times of MDA-MB-231, SK-BR-3, and MCF-7 according to exponential fits in Fig. 2a are 10.6 ms, 16.2 ms, and 27.4 ms, respectively. Figure 2b shows the average entry time for all cell sizes is also shorter for the MDA-MB-231 cell than it is for the other two cell lines.

This confirms that the entry time can be a good index to measure the mechanical deformability of cancer cells with various levels of invasiveness^13,14^. Figure 2c illustrates the average entry time for various cell sizes (ranging from 13–21 μm) of the three cell lines. This figure indicates the difference in the deformability of the three cell lines becomes visible for cell sizes greater than 17 μm. The results in Fig. 2 demonstrate higher deformability of MDA-MB-231 cell line compared to the other two cell lines consistent with the studies that reported it to be capable of metastasizing in vivo when it is directly injected to the circulatory system^57^.

**Fig. 3:**
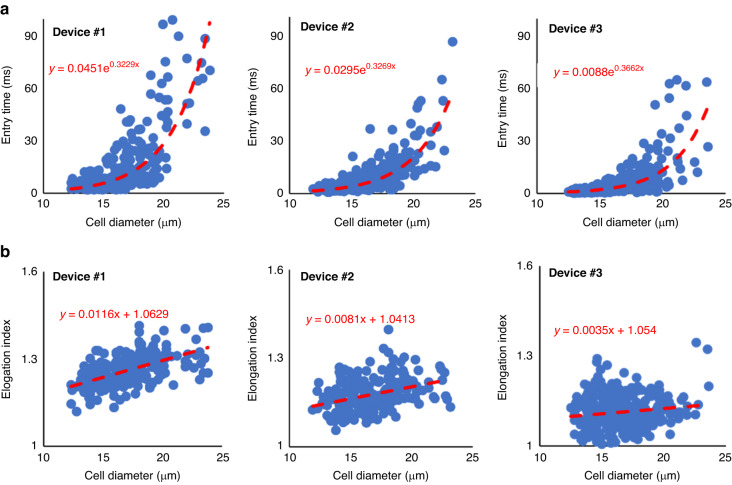
Deformability measurement of MDA-MB-231 using the three microfluidic devices. Experimental measurement of the deformation behavior of the highly metastatic breast cancer cell line (MDA-MB-231) using the three microfluidic devices by measuring **a** the entry time of single cancer cell passing the constricted channels, and **b** the elongation index

Because the deformation behavior of MDA-MB-231 cells entering a narrow channel is closer to that of CTCs passing confined microcapillaries than the other two cell lines, we focused on expanding the deformability measurements of this cell line to also use the data for developing validated SNMs of breast cancer cells. More specifically, we measured the entry time and elongation index of MDA-MB-231 passing through the constricted channel of all three microfluidic devices fabricated in this study. Figure 3 shows the measured entry time for different cell sizes as well as exponential fits for the three devices. The data in Fig. 3 show that for a fixed cell diameter, the entry times and the elongation indexes of the cell passing a wider constricted channel are smaller. For instance, the fitted curves in Fig. 3a show the entry time for an 18 μm MDA-MB-231 that was measured using Device #1, Device#2, Device#3 is 15.1, 10.6, and 6.4 ms, respectively. In addition, the elongation indexes of 18 μm MDA-MB-231, calculated from the fitted curves in Fig. 3b, are 1.27, 1.18, and 1.11 using Device #1, Device #2, and Device #3, respectively.

### Parameter identification and numerical results

**Fig. 4:**
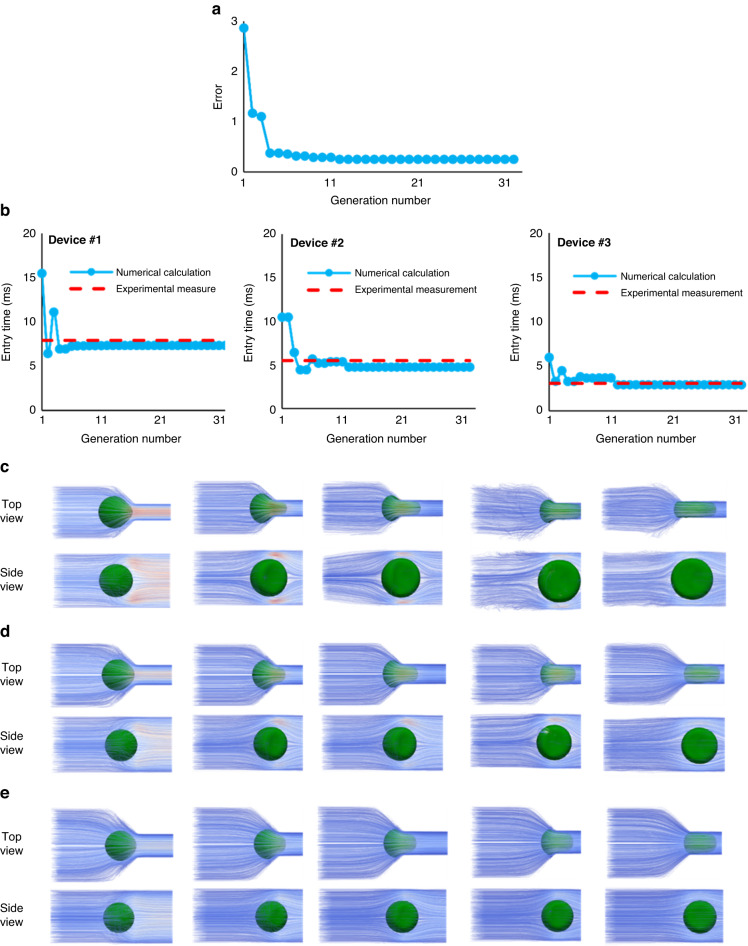
Cell model parameter identification of the 16 μm cell using GA. **a** Error minimization for the cell entry time completed after 32 successive generations. **b** Entry time convergence to the experimental values in the three devices shown at every generation. **c**–**e** The numerical simulations of the entry process of the cell entering the constricted channels after applying the identified parameters for the cell model in the three devices illustrated at five instances in two different views. (Supplementary information Video 2 to Video 4)

The deformability of the cells can be captured using the SNM if the correct model parameters are identified. Using the GA, as described in Materials and Methods, we identified the unknown model parameters utilizing the fitted curves to the measured entry times of MDA-MB-231 for 6 different cell sizes between 13 μm to 18 μm in the three microchannel devices (in Fig. 3). In this study, for each cell size the identification procedure was performed using the experimental data of that cell size separately to increase the identification accuracy. For the MDA-MB-231 cancer cell with the diameter of 16 μm, the evolution of the error function (Eq. 11) considering all three microchannel devices for the best set of parameters at every generation is shown in Fig. 4a. The numerically calculated entry times in each of the three microchannel devices using this GA process are shown in Fig. 4b. As Fig. 4a shows, for this cell size, the error got close to an optimum value after four generations, improved between generations 5–12, and remained unchanged after the 12th generation. Figure 4b shows that convergence of the entry times follows the same trend as the error function in Fig. 4a.

Figure 4c–e shows five instances of the simulated entry process of the 16 μm cell after applying the identified quantities for the unknown parameters of the cell model in three devices. The time instances in these panels are different but were selected to show comparable squeezing state between the three devices. The shape of the cell and its interaction with fluid and microfluidic walls are illustrated from two different views as the cell enters the constricted channel in each microfluidic device. The streamlines in these figures visualize the fluid flow interactions with the squeezing cell and the walls of the devices. Streamlines experience the greatest disturbance in Device #1, where the greatest size mismatch between the cell and

constricted channel sizes exists. The disturbance is the most visible in the two last time instances (two rightmost) of Fig. 4c when the cell is near to complete entry to the constricted channel. The same cell size causes a minimal disturbance in the streamlines in Device #3 close to the complete entry state (two leftmost states in Fig. 4e). Supplementary Videos 2 to 4 show the squeezing process of Fig. 4c–e with a higher temporal resolution. The parameter values, that reached the best rank at each generation for 16 μm cells, are given in Table 1. Moreover, the results of the identification step including the identified parameter values and the minimized error of every cell size are summarized in Table 2.

**Table 1:** The quantities of the 16 μm cell model parameters for the best ones at each generation

Generation	${K}_{b}$	${K}_{v}$	${K}_{a}$	${K}_{l}$	${VR}$	${E}_{1}$	${E}_{2}$	${E}_{3}$	${\rm{Error}}$
1	79,675	34,530	31,834	80,957	1.2	0.960911	0.905116	0.94585	2.811876
2	84,715	49,604	46,274	95,927	2.2	0.185705	0.905116	0.081388	1.172209
3	53,904	49,983	9980	18,070	16.9	0.40797	0.170537	0.464981	1.104359
4	383	70,593	33,096	20,175	13.8	0.120177	0.191038	0.06939	0.380605
5	383	70,593	33,096	20,175	13.8	0.120177	0.191038	0.06939	0.380605
6	52,948	23,342	1014	30,366	10.2	0.081467	0.0332	0.247082	0.361749
7	65,639	24,273	35,053	18,419	19.9	0.07704	0.050798	0.192931	0.320769
8	65,639	24,273	35,053	18,419	19.9	0.07704	0.050798	0.192931	0.320769
9	45,361	92,626	20,013	22,461	19.2	0.072106	0.021771	0.20201	0.295887
10	45,361	92,626	20,013	22,461	19.2	0.072106	0.021771	0.20201	0.295887
11	45,361	92,626	20,013	22,461	19.2	0.072106	0.021771	0.20201	0.295887
12	45,526	93,182	19,816	23,203	19.9	0.070715	0.132257	0.050584	0.253555

**Table 2:** The quantities of the identified parameters for various cell sizes ranging from (13–18 μm)

Cell diameter (μm)	${K}_{b}$	${K}_{v}$	${K}_{a}$	${K}_{l}$	${VR}$	${\rm{Error}}$
13	98,769	90,341	10,028	22,644	3.8	0.21
14	96,514	47,006	15,056	18,458	14.9	0.17
15	64,763	45,165	79,571	8854	39.9	0.30
16	45,526	93,182	19,816	23,203	19.9	0.25
17	52,542	110	19,948	29,936	37.2	0.51
18	12,109	1420	6958	33,000	21.9	1.07

**Fig. 5:**
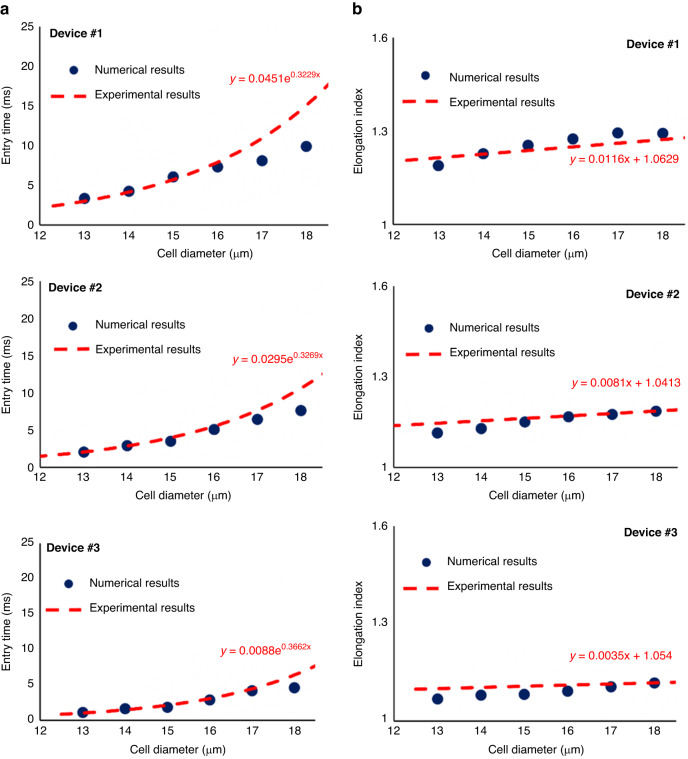
Comparison between numerical results with the curve fitted on the population of the experimental data. The comparison between experimental results and the numerical ones for **a** the entry time, and **b** elongation index of MDA-MB-231 cell line taken after applying the identified parameters of the cell model

The model should be able to replicate the motion and deformation of the population of cells observed experimentally. To verify this, we quantified the numerically calculated entry time and elongation index of the cell after complete entry and compared them with ones obtained experimentally for the population of MDA-MB-231 cells in all three devices represented by the fitted curves in Fig. 5 (reproduced from Fig. 3).

The numerical calculation used the identified parameters reported in Table 2. Figure 5 shows that the numerically obtained entry times and the shape of the cells (characterized by the elongation index at full entry to the constriction) closely replicate the motion and deformation of the population of the cell observed experimentally. The average errors among all devices and cell sizes were 13% for the entry time and 1.4% for the elongation index at full entry to the constriction.

**Fig. 6:**
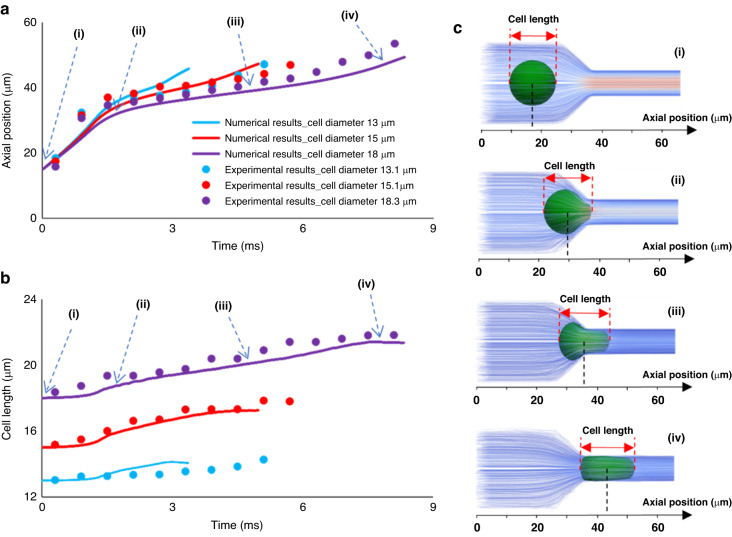
Comparison between numerical results and experimental ones for cell deformation behavior of three distinct data. The comparison between experimental results and the numerical ones for **a** axial position, and **b** cell length of three MDA-MB-231 cells with different size during their entry into constricted channel of device #2. (Supplementary Video 5 to Video 7) **c** Descriptive images of cell length change and cell axial position during the entry process

Moreover, in Fig. 6, the numerical and experimental results of the axial cell position and evolution of the cell length during the entry process of MDA-MB-231 in Device #2 were compared. In this figure, data for cell diameters of 13, 15 and 18 µm are presented. Figure 6a shows that increasing the cell diameter makes the cell squeezing into the constriction longer and this behavior was consistently observed in experimental and numerical data. Furthermore, the validated numerical model closely reproduced the experimental observations. Figure 6b shows the gradual and consistent increases in the cell length calculated from the numerical model are very similar to that of the experiment for all three cell sizes. In addition, these observations can also be seen in similarly, Supplementary Videos 5–7 show squeezing of cancer cells into the microchannel, increasingly decelerates the cell motion with increasing the diameter from 13 to 18 µm in the same constricted channel (Device #2). Figure 6c illustrates the axial cell position and cell length at 4 different time steps during cell entry.

**Fig. 7:**
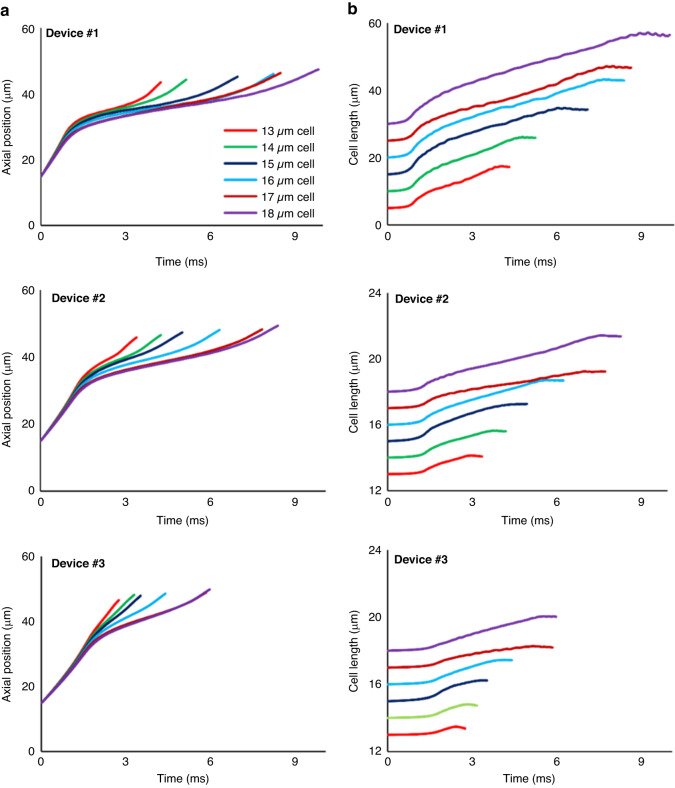
The motion and deformation behavior of MDA-MB-231 cells using the validated numerical models. Detailed investigation of motion and deformation behavior of MDA-MB-231 cells was performed by using the developed numerical models of passing the single cells through the constricted channel in the three constricted devices. The numerical model could calculate the shape changes of the cell in terms of **a** the cell position and **b** the cell length at each time step of the gradual squeezing of the cell into the constricted channels

**Fig. 8:**
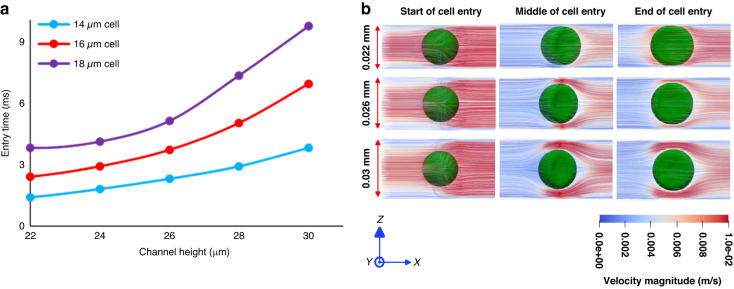
Numerical investigation of channel height effect on cells’ entry times. **a** The numerical results of the cancer cells entry times entering the 10 µm constricted channel with various heights at 20 µL/h fluid flow rate using the developed cell models. **b** The side view images of the numerically simulated entry process of the cell entering the 10 µm constricted channels with the channel height 22, 26, 30 µm at start, middle, and end of the entry process

We used this validated model to obtain the deformation behavior of the cancer cell in terms of cell length and cell center trajectory using all identified cell model with different sizes (Table 2) that are numerically investigated in detail in Fig. 7. This figure shows that increasing the cell size within one device increasingly decelerates the cell squeezing making the entry time increasingly longer. As an example, in Device #1, the entry time goes up from 4 ms to 10 ms when the cell diameter changes from 13 µm to 18 µm. On the other hand, increasing enlargement of the constricted channel from Device #1 to Device #3 (from 8 µm to 12 µm) increasingly shortens the entry time from the maximum 10 ms for 18 µm cell in Device #1 to ~6 ms for the same cell size in Device #3. Figure 7b shows that the most drastic cell-shape change (5 µm increase in the cell length) occurs with the most cell-channel size mismatch (18-µm-diameter cell in 8-µm channel of Device #1). Consistently, the least cell-shape change (0.5 µm increase in the cell length) occurs with the least cell-channel size mismatch (13-µm-diameter cell in 12-µm channel of Device #3). Within one channel size, increasing the cell diameter increases the cell deformation. For example, in Device #1, cells of diameters 13 µm and 18 µm experience 3.5 µm and 5.3 µm length change, respectively. In addition, using the validated 3D cell models, the effect of changes in the channel height on the entry time of cells with different sizes was investigated (Fig. 8). As Fig. 8 illustrates, for the same fluid flow rate (20 µL/h) flowing through the 10 µm width constriction, increasing the channel height decreases the fluid velocity in the channel, and as a result the cell entry time nonlinearly increases for all three cell sizes. As an example, for the 16 µm cell, increasing the channel height from 22 µm to 30 µm caused the entry time to increase nonlinearly from 2.4 ms to 6.9 ms. Figure 8b shows the side view of the entry process of 18 µm cancer cell at three instances (start, middle and end of cell entry) entering the constricted channel of device #2 with various channel height. The physical time of these shown instances were different, but all are captured at the comparable instant of the entry process. As this figure shows, the decrease in the fluid velocity magnitude due to increasing the channel height can be noticed specially at the back and front of the cell that illustrates the decrease in cell velocity.

**Fig. 9:**
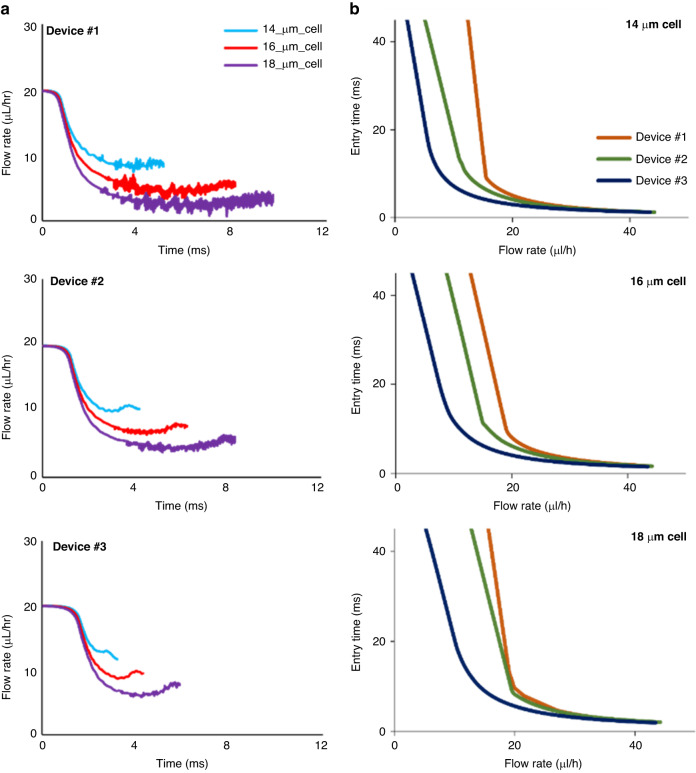
Numerical investigation of fluid flow rate change during cell entry process and the effect of fluid flow rate on cells’ entry time. **a** Change of the flow rate while the cell entering the channel that was calculated numerically indicates flow rate change dependency to the cell size. **b** The effect of fluid flow rate on the cell entry time of various cell sizes in the three different constricted channels was investigated numerically

Since in the present model the FSI is a two-way communication between the cell and fluid at each time step^58^, the model is capable of calculating the time-dependent effect of the cell entry process on the fluid flow rate in the microchannel. In fact, capturing the effects of the fluid motion on the cell deformation can help unraveling the impact of hemodynamics on the hematogenous spread of metastasis^6^. Figure 9a shows the changes in the flow rate as a cell squeezes in the constricted channels of the three devices for three cell sizes. In all devices and cell sizes shown in Fig. 9a, the initial flow rate of 20 µL/h starts to drastically drop as cell squeezing starts and progresses but the rate and magnitude of the drop depends on the cell and microchannel sizes. For example, for the cell diameter of 18 μm in Device #1 the flow rate decreases to 0.7 μL/h while the lowest flow rate in this device for 16 μm cell and 14 μm cell are 3.3 μL/h and 7.4, respectively. Besides, the constricted channel width affects the fluid flow rate decrease during the entry process as for 18 μm cell in Device #2 and Device #3 the flow rate reaches as low as 3.8 μL/h, and 5.8 μL/h, respectively.

Figure 9b shows the effect of the flow rate on the entry time of the cell. For the flow rates more than 40 µl/h, the entry times of three different cell sizes (14, 16, 18 µm) are almost the same in the constricted channel of all three devices. For instance, the numerically calculated entry times for cell diameters of 14, 16, and 18 μm that pass the constriction in Device #1 with 40 μL/h flow rate are 1.4 ms, 1.8 ms, and 2.3 ms, respectively. As the flow rate decreased from 40 μL/h to lower values, the effects of the cell and microchannel sizes become increasingly more remarkable. For example, in Device #1 with the flow rate of 20 μL/h the entry times of 4.2, 7.3, and 9.6 ms were calculated. However, in Device #3 at the flow rate of 10 µl/h the entry times of cells with the diameters of 14 µm and 18 µm was calculated to be 6.1 ms and 18.3 ms, respectively.

## Conclusion

This study investigated the feasibility of numerically replicating the deformation behavior of single CTCs passing confined spaces in microcapillaries using an advanced in-silico method at the cellular scale. The proposed SNM-based in-silico method was enabled by the previously developed optimization code that encapsulates the experimental data in a validated model of cancer cell deformation. For simplicity, this study used experimental deformation of cancer cell lines in vitro in constricted microfluidic devices instead of studying real CTC deformations in real human microcapillaries. In the first step of this study, the deformability of three different breast cancer cell lines (MCF-7, SK-BR-3, and MDA-MB-231) was measured. The results show the highly metastatic cell line (MDA-MB-231) is the most deformable one among the three confirming this cell line merit for using as CTCs’ replica in the in-vitro experiments. Therefore, the optimization algorithm for parameter identification of the cell membrane model was applied to the experimental results of the deformability of highly metastatic breast cancer cells (MDA-Mb-231) passing constricted microfluidic devices with various widths of the constricted channel (ranging from 8 to 12 μm). The parameter identification step, which was performed on various cell sizes (ranging from 13 to 18 μm), helps achieve accurate results for the deformation behavior of discrete cell models in a range

of cell diameters. The numerical results show good agreements with the experimental ones in terms of both the entry time and elongation index in various geometrical domains. This means that the results of the numerical models are valid for both the gradual squeezing of the cell into the constrictions and the shape of the cell during the entry process. To the best of our knowledge, this study is the first one to present a valid discrete numerical model for the deformation behavior of highly metastatic cancer cells (MDA-MB-231) for a range of cancer cell diameters. The agreement between numerical and experimental results for large deformation of highly metastatic cancer cells opens doors for further investigations of complex biological phenomena that are instrumental in the hematogenous spread of metastasis with the use of the proposed method. Since the mechanical properties of each cancer cell type is different from other cancer cell types, the combined experimental-numerical method, proposed in this work, can be used to obtain the valid model specific to that cell type. Such models can be used to investigate the situation in which cancer cells physically occlude a microcapillary or adhere to a vessel wall can be studied by applying the validated model presented here. Furthermore, the motion and deformation behavior of CTC clusters can be numerically obtained by repeating the presented approach for CTC clusters. Finally, measuring CTCs deformability acquired from the liquid biopsy in patients and developing the numerical model based on the deformability of real CTC data can advance the numerical model to a higher level for potential future clinical applications.

## Materials and methods

### Cancer cells culture

Immortalized breast cancer cell vials (MCF-7, SK-BR-3, and MDA-MB-231 cell lines with moderate to high metastatic potential^65^) were taken out from storage and cultured in Dulbecco’s Modified Eagle Medium, DMEM, (4.5 g/L glucose, with L-glutamine & phenol red without sodium pyruvate, WISENT Inc.) supplemented with 10% v/v of fetal bovine serum (WISENT Inc.) and 1% v/v of streptomycin (WISENT Inc.) in 75 cm^2^ T-flasks (Thermo Fisher Scientific). Cells were incubated in a standard humidified incubator at 37 °C and 5% CO2. Cells upon reaching a confluency of more than 70% were detached and passaged. On the day of the experiment (half an hour before the experiments), cells were detached with 0.25% trypsin-EDTA (WISENT Inc.) after three or less passages. Cells were quantified for their viability with trypan blue with an automated cell counter (Olympus Life Science). Two to four million cells per mL were grown and used for every experiment.

### Design, fabrication, and characterization of microfluidic devices

**Fig. 10:**
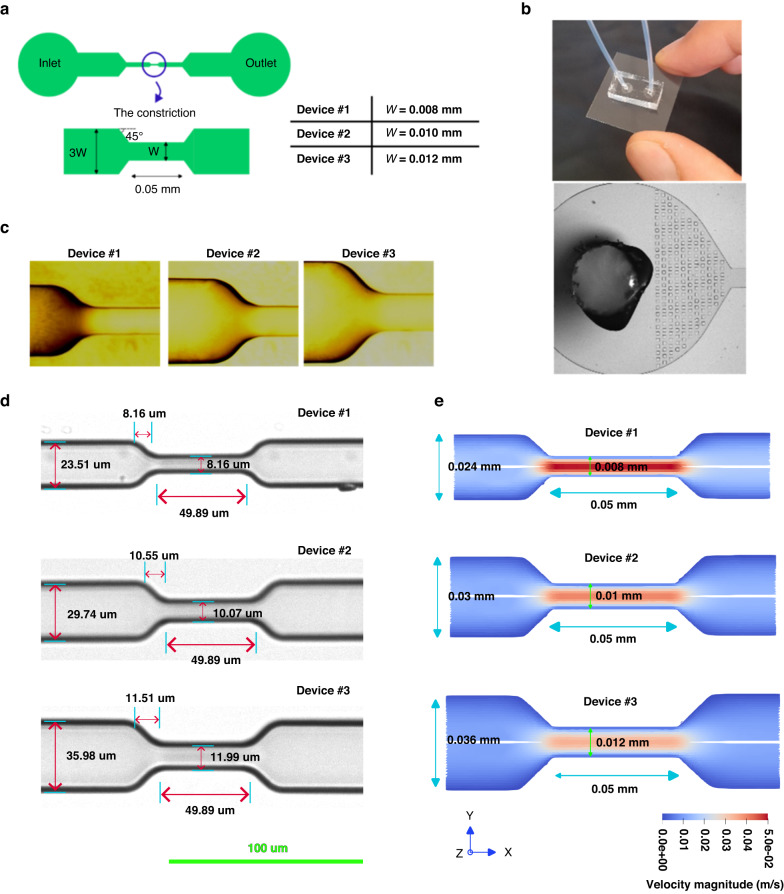
Design and characterization of the constricted microfluidic devices accompanied by calculated fluid velocity of the numerical domain. **a** Schematic representation of the microfluidic devices design. **b** Actual image of the designed one-channel device and the filters devised in the inlet. **c** Magnified view of the entrance section of the constricted channels in the fabricated mold using 100× objective. **d** Magnified view of the constricted channel in the fabricated devices using 20× objective. **e** The fluid velocity magnitude at the mid-plane in the Z direction for 20 μL/h flow rate extracted from 3D CFD simulations of each constricted channel

The design of microfluidic devices contains a single constricted channel in the middle with sizes comparable to human microcapillaries to assure cell deformation at the constrictions’ entrance (Fig. 10a, b). The microfluidic devices were fabricated using soft lithography techniques. One layer master mold was fabricated by spin coating SU-8 photoresist 2015 (Kayaku Advanced Materials) at 1400 rpm for 30 s on a silicon wafer. After spin-coating, the wafer was soft baked at 65 °C for 1 min followed by 95 °C for 4 min, and 65 °C for 1 min. Then, the photoresist was exposed to UV light using Karl Suss MA6 Mask Aligner through a previously designed and fabricated chromium glass mask (Nanofab, Alberta University) for 6 s. Next, the post-exposure bake was performed similar to the soft bake procedure. Then, the wafer was developed for 5 min with a SU-8 developer and located on a hot plate at 150 °C for 20 min to stabilize the SU-8 microstructures. The microscopic images of the constricted channels patterned in the master mold are shown in Fig. 10c. In addition, the height of the microchannels was measured to be 28 μm using a surface profilometer (Dektak 8 Stylus Profilometer). After fabricating the silicon master mold, polydimethylsiloxane (PDMS) monomer and curing agent were mixed at a 10:1 volumetric ratio. Then, the mixture was degasified in the desiccator, poured on the silicon master, and thermally cured at 70 °C for 2 h. The cured PDMS was stripped off from the silicon master. Then, the cured PDMS containing the constricted microchannel replicas were cut to the appropriate size, punched in the inlets and outlets to obtain the fluidic access holes, and bounded to a glass slide using oxygen-plasma bonding. The device fabrication was completed by connecting silicon tubing secured with glue to the fluidic access holes. To characterize the critical features of the microfluidic devices, bright field images of the constricted channel of all devices captured by an inverted microscope (Nikon Ti Eclipse) were analyzed. Using a 20× objective, magnified images of the constricted channels, constriction entrance, and exit portion were captured and measured. As Fig. 10a, d show three constricted devices each of which has a 45° tapered entrance at the constricted channel, whose width is comparable to microcapillary diameters ranging from 8 to 12 µm^34^, that fabricated and used in this study. Moreover, comparing to the constricted channels used in the literature^16,17,38^, measuring cell deformability with cell diameter ranging from 13 µm to 26 µm, the fabricated constricted channels in the present study made measuring the single-cell deformability of almost all sizes of the targeted cancer cells feasible. In each device, the width of the channel before the constricted channel is 3 times larger than the width of the constricted channel. This assures both the device fabrication with accurate features and keeping the numerical domain small enough for performing the parameters identification. It is worth mentioning that cell samples were not filtered prior to infusing into the microfluidic devices, but square shape filters with 50 µm distance from each other were devised in the inlet of each device to help reducing cell aggregate at the constricted channel (Fig. 10b).

### Numerical method

#### Lattice Boltzmann method (LBM)

The entry process of a single cancer cell into the constricted channels was modeled using Hemocell open-source code (version 2.4)^57^. In this code, the fluid is considered as an incompressible Newtonian fluid whose motion is described in a Eulerian framework and solved by Lattice Boltzmann Method (LBM) implemented in Palabos open-source code (version 2.0)^66^. More specifically, a three-dimensional 19-velocity cube lattice scheme (D3Q19) is utilized in the LBM governing equations as follows:

1 $$\begin{array}{l}{n}_{i}\left(x+{e}_{i}\varDelta t,t+\varDelta t\right)={n}_{i}\left(x,t\right)-\frac{1}{\tau }\left({n}_{i}\left(x,t\right)\right.\\ \left.-\,{n}_{i}^{{eq}}\left(x,t\right)\right)+{f}_{i}\left(x,t\right)\,{\rm{for}}\,i=1,2,\ldots ,19\end{array}$$

where ${n}_{i}\left(x,t\right)$, ${e}_{i}$, $\varDelta t$, $\tau$ and ${f}_{i}\left(x,t\right)$ are the density distribution function, the velocity vector, time step, relaxation time toward the equilibrium distribution ${n}_{i}^{{eq}}$, and external force, respectively. At each lattice

site, the macroscopic fluid density ρ and velocity u can be obtained from the particle density functions as follows:

2 $$\begin{array}{l}\rho \left(x,t\right)=\mathop{\sum}\limits_{i}{n}_{i}\left(x,t\right)\,\,{\rm{for}}\,i=1,2,\ldots ,19\\ \rho \left(x,t\right)u=\mathop{\sum}\limits_{i}{n}_{i}\left(x,t\right){e}_{i}\,\,{\rm{for}}\,i=1,2,\ldots ,19\end{array}$$

The numerical domains of the three constricted microfluidic devices (shown in Fig. 10a, d) have been created and meshed in a CAD software (Salome 9.7.0))^67^ and the fluid passing through each microchannel was modeled using the above described LBM implementation in Hemocell and Palabos. Figure 10e shows fluid flow simulation at the mid-plane along height direction (z-axis) in three microfluidic devices. Moreover, both the channels’ geometry and the geometrical domain in x-y plane that used for numerical simulation of the cell entry process in the constricted devices were shown in Fig. 10e.

#### Cell Membrane Model

The cancer cell is considered as a membrane with a spherical shape which is discretized by two-dimensional triangles with springs on the triangles’ edges. The constitutive equations governing the deformation behavior of the cancer cell include a set of forces (the link force, the bending force, the local area force, and the volume force) acting on the cell membrane as described below^57^.

The link force acts along the edge that connects two adjacent cell membrane vertices is representing the stretch force on the vertices as defined below:

3 $${F}_{{\rm{link}}}=-\frac{{K}_{l}{k}_{B}T}{P}\frac{({L}_{i}-{L}_{0})}{{L}_{0}}\left[1+\frac{1}{{\tau }_{l}^{2}-{(\frac{{L}_{i}-{L}_{0}}{{L}_{0}})}^{2}}\right]$$

where ${K}_{l}$, ${k}_{B}$, ${\rm{T}}$, ${L}_{i}$, ${L}_{0}$ are the link modulus, the Boltzmann constant, temperature, the current length of the edge, and the initial length of the edge, respectively. $P=7.5\,{\rm{nm}}$ is the persistence-length of the edge, and ${\tau }_{l}=3$ is the relative expansion ratio at which the edge reaches its persistence length^57^.

The bending force is defined in terms of the change in the angles between the normal vectors of two adjacent surface elements as follows:

4 $${F}_{{\rm{bend}}}=-\frac{{K}_{b}{k}_{B}T({\theta }_{i}-{\theta }_{0})}{{L}_{0}}\left[1+\frac{1}{{\tau }_{b}^{2}-{({\theta }_{i}-{\theta }_{0})}^{2}}\right]$$

where ${K}_{b}$, ${\theta }_{i}$, and ${\theta }_{0}$ are the bending modulus, current and initial angles between the normal vectors of the surface elements, respectively. ${\tau }_{b}$ is the limiting angle and is chosen to be $\frac{\pi }{6}$ to prevent unrealistic sharp surface edges^57^.

The local area force applies on each surface element vertices and represents the reaction of the element to change of its area as follows:

5 $${F}_{{\rm{area}}}=-\frac{{K}_{a}{k}_{B}T}{{L}_{0}}\frac{{A}_{i}-{A}_{0}}{{A}_{0}}\left[1+\frac{1}{{\tau }_{a}^{2}-{(\frac{{A}_{i}-{A}_{0}}{{A}_{0}})}^{2}}\right]$$

where ${K}_{a}$, ${A}_{i}$, and ${A}_{0}$ are the area modulus, the current and the initial area of the triangle, respectively. ${\tau }_{a}=0.3$ is the area limiting factor to prohibit surface area changes more than 30%^57^.

The global volume force applies on all vertices of the cell membrane and conserves the volume of the cell.

6 $${F}_{{\rm{volume}}}=-\frac{{K}_{v}{k}_{B}T}{{L}_{0}}\left(\frac{{V}_{i}-{V}_{0}}{{V}_{0}}\right)\left[\frac{1}{{\tau }_{v}^{2}-{(\frac{{V}_{i}-{V}_{0}}{{V}_{0}})}^{2}}\right]$$

where ${K}_{v}$, ${V}_{i}$, and ${V}_{0}$ are the volume modulus the current and the initial volume of the cell membrane, respectively. ${\tau }_{v}=0.01$ is the volume limiting factor to resist changes in the cell volume^57^.

**Fig. 11:**
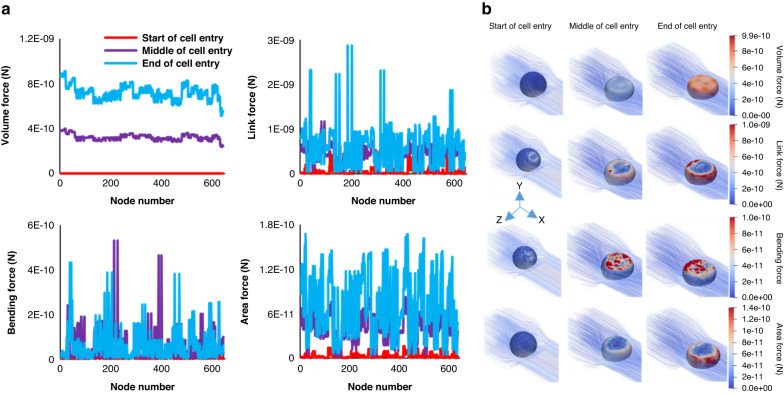
Magnitude of forces acting on the cell membrane during cell entry. **a** Magnitude of cell model forces acting on every node of the cell extracted at three different instances (start, middle, end) during cell entry process for the 18 µm cell entering the constricted channel of device #2. **b** Descriptive images of the cell model forces contributing to entrance of the cell into the constriction at the same instances as part (**a**)

It worths noting that the cell model used in this study consists of 642 nodes on which all the mentioned forces have been applied at every time step. Figure 11 illustrates the magnitude of the forces at three instances of cell entry process (start, middle, end) for 18 µm cancer cell model entering the constricted channel of device #2. This figure shows all forces are at their minimum value before the cell deformation starts. For the most or all the vertices the values of the Volume forces, the Link forces, and the Area forces increase as the cell is squeezing to the constriction and reach to their maximum value at the end of the entry process. For the bending forces maximum values were reached during the cell squeezing. Figure 11b depicts the forces act on the cell membrane nodes at the mentioned instances by outputting the cell model during the entry process.

To achieve a realistic numerical model in this study, the internal viscosity of the cell was assumed to be different from the exterior fluid. Therefore, a dimensionless parameter named Viscosity Ratio (VR) was considered as follows:

7 $${\rm{VR}}=\frac{{{\rm{Interior}}}\,{\rm{cell}}\,{\rm{fluid}}\,{\rm{viscosity}}}{{{\rm{the}}}\,{\rm{fluid}}\,{{\rm{viscosity}}}}$$

Therefore, ${K}_{l}$, ${K}_{b}$, ${K}_{a}$, ${K}_{v}$, and ${VR}$ are dimensionless parameters that need to be identified accurately to enable the numerical model to replicate the deformation behavior of the cancer cell captured in the correspondence experiment.

#### Fluid-solid interaction (FSI)

Here, fluid-cell interactions were modeled with Immerse Boundary Method (IBM) which acts as a bridge between Eulerian grids of the fluid and Lagrangian grids of the cell membrane^68^. More specifically, the exerted forces on the cell membrane nodes, determined by the cell’s constitutive equations $(F)$, were spread on the fluid grids as follows:

8 $$f\left(x,t\right)=\int F\left(q,t\right)\delta \left(x-X\left(q,t\right)\right){dq}$$

where $\delta$ is the Dirac delta function, $x$ is the coordinate of the Eulerian grids, and $X(q,t)$ is the position of a cell node with Lagrangian coordinate $q$ at time $t$.

The velocity of the cell membrane nodes $U\left(X\left(q,t\right)\right)$ was obtained from the integral below and applied for updating the positions of the nodes.

9 $$U\left(X\left(q,t\right)\right)=\int u(x,t)\delta \left(x-X\left(q,t\right)\right){dx}$$

where $u\left(x,t\right)$ is the velocity of the fluid with Eulerian grid $x$ at time $t$.

#### Cell–wall interaction

Furthermore, repulsive forces were defined between the nodes of the cell and microchannel wall to avoid cell penetration into the microchannel wall to model the behavior of the cell near the walls as:

10 $${\vec{F}}_{r}\left(d\right)={\kappa }_{{rep}}\frac{{d}_{{cut}}}{d}\vec{m},d \,<\, {d}_{{cut}}$$

where ${\kappa }_{{rep}}$ is the repulsion constant, $d$ is the distance between the nodes of cell and wall, ${d}_{{cut}}$ is the threshold of repulsive force activation, and $\vec{m}$ is the unit vector pointing from the wall node to the cell node^57^. The repulsive-force parameters were constant in all simulations in this study (Table 3).

**Table 3:** Parameters used in this study

Parameters	Symbol	value	Unit
Time step	$\varDelta t$	0.01	μs
Lattice resolution	$\varDelta x$	1	μm
Fluid kinematic viscosity	$\upsilon$	1.1 × 10¯^6^	$\frac{{{\rm{m}}}^{2}}{\rm{s}}$
Fluid density	$\rho$	1025	$\frac{{\rm{kg}}}{{\rm{m}}^{3}}$
Repulsive force activation threshold	${d}_{{cut}}$	0.8	μm
Repulsive force scale coefficient	${\kappa }_{{rep}}$	0.004	-

### Genetic algorithm (GA)

The mentioned parameters which describe the deformation behavior of the cancer cell were identified using the previously developed genetic algorithm^8^. This algorithm benefits from creating the first population of size 60 using randomly generated multi-digits binary numbers. Every 128 digits of the binary numbers represents one of the parameters and each row of the first population represents a set of parameters ${K}_{l}$, ${K}_{b}$, ${K}_{a}$, ${K}_{v}$, and ${VR}$. Afterwards, every two rows of the first population were selected as parents and crossover has been performed for the crossover probability higher than a user-defined probability (0.8) for generating children. Then, mutation step was conducted for the mutation probability higher than a user-defined probability (0.6) on every digit of the binary numbers that made by crossover. The made children and the mutated ones were added to the previously generated first population. Then, the binary numbers were converted to decimal numbers according to the upper and lower bounds of each parameter as provided in Table 4. Then, using the decimal numbers, numerical simulations of the cancer cell entry process were performed in all three devices benefiting from parallel jobs on supercomputers. It is worth noting that every generation consisted of 120 sets of parameters, therefore, 360 simulations were performed simultaneously using 2 cores for each and the entry time was stored when the cells fully entered the constricted channels. For those simulations, that the entry process took much longer than the experimental data, the simulations were stopped when the entry time reached a user-defined value (twice of the experimental data). Finally, the outcomes of the numerical simulations and the experimental data were compared based on the below error function:

11 $${\rm{Error}}=\mathop{\sum }\limits_{n=1}^{{n}_{t}}{E}_{n}=\mathop{\sum }\limits_{n=1}^{{n}_{t}}\left|1-{\left(\frac{{{{\rm{ET}}}}^{s}}{{{{\rm{ET}}}}^{e}}\right)}_{n}\right|$$

where ${E}_{n}$,$\,{{\rm{ET}}}^{s}$, and ${{\rm{ET}}}^{e}$ are the error in the *n*th device, numerically calculated entry time, and experimentally measured entry time, respectively. Here, ${n}_{t}$ is equal to 3 for using the entry time at three different constricted devices.

**Table 4:** Upper bound and lower bound for cell model parameters

	${K}_{b}$	${K}_{v}$	${K}_{a}$	${K}_{l}$	${VR}$
Lower bound	0	0	0	0	0
Upper bound	10^6^	10^6^	10^6^	10^6^	40

At the end, the results for the parameters of the cell constitutive equations were sorted and the best 20 ones added to the initial population of the next generation. The algorithm stops if the error remains unchanged after 20 successive generations.

### Experimental setup

Before running the experiments, the devices were degassed for up to one day with Pluronic solution to avoid cell adhesion to the channels and washed with a constant flow of PBS for 20 min. The microfluidic device was placed on the stage of a Nikon Ti Eclipse inverted microscope in the experimental setup shown in Fig. 1a. The cell sample flowing in the media consisting of RPMI-1640 solution with 20% fetal bovine serum (FBS) (the media density $\rho =1020\pm 5\frac{{{\rm{kg}}}}{{{\rm{m}}}^{3}}$, and the media dynamic viscosity $\mu =1.089\pm 0.044\,{{\rm{mPa}}}\,{\rm{s}}$)^69^ with cell viability of %90 or higher was infused into the constricted channel using a syringe pump (Chemyx Inc., USA) at a constant flow rate of 20 μL/h. Cell samples with the concentration of 2 × 10^6^ cells per mL of media were prepared and used for all experiments. In addition, since the main focus of the present study is to validate the numerical model of single cancer cell deformation behavior, only the data of single cancer cells that pass the constricted channels one at a time have been gathered. More specifically, the captured data of cell clusters and more than one cells in the constricted channels at the same time were set aside from further analysis. At this flow rate, the entry time measured for the average cell size is less than 16 ms. The flow of cancer cells into the constricted channels has been visualized in bright field mode of the inverted microscope using a ×20 magnification objective and recorded using a high-speed camera (FASTCAM S1 model, Photon USA, Inc.) at a high frame rate of 5000 fps with the spatial resolution of 512 × 512 pixels. All captured videos and images were analyzed manually using Photron Fastcam Viewer 4 (PFV4) software to measure cell size, entry time, and elongation index which is the ratio of cell length after entering the constriction to the original cell length. Figure 1b–d shows MDA-MB-231 cell squeezing and entering the microchannel sizes of 8, 10 and 12 μm, respectively, at five instances until completion of cancer cell entry.

### Supplementary information

Supplementary Videos

## References

[1] Wirtz D, Konstantopoulos K, Searson PC (2011). The physics of cancer: the role of physical interactions and mechanical forces in metastasis. Nat. Rev. Cancer.

[2] Krog, B. L. & Henry, M. D. Biomechanics of the circulating tumor cell microenvironment. in *Biomechanics in Oncology,* 1092 (eds. Dong, C. et al.) 209–233 (Springer International Publishing, 2018). 10.1007/978-3-319-95294-9_11 PMC7304329 30368755

[3] Ewing, J. Neoplastic diseases: a treatise on tumours. By James Ewing, A.M., M.D., Sc.D., Professor of Pathology at Cornell University Medical College, N.Y.; Pathologist to the Memorial Hospital. Third edition. Royal 8vo. Pp. 1127, with 546 illustrations. 1928. Philadelphia and London: W. B. Saunders Co. Ltd. 63s. net. *Br. J. Surg.* **16**, 174–175 (1928).

[4] Weiss L, Bronk J, Pickren JW, Lane WW (1981). Metastatic patterns and target organ arterial blood flow. Invasion Metastasis.

[5] Follain G (2018). Hemodynamic forces tune the arrest, adhesion, and extravasation of circulating tumor cells. Dev. Cell.

[6] Follain G (2020). Fluids and their mechanics in tumour transit: shaping metastasis. Nat. Rev. Cancer.

[7] Poudineh M (2017). Tracking the dynamics of circulating tumour cell phenotypes using nanoparticle-mediated magnetic ranking. Nat. Nanotechnol..

[8] Keshavarz Motamed P, Maftoon N (2021). A systematic approach for developing mechanistic models for realistic simulation of cancer cell motion and deformation. Sci. Rep..

[9] Xiao, L. L., Yan, W. W., Liu, Y., Chen, S. & Fu, B. M. Modeling cell adhesion and extravasation in microvascular system. In *Molecular, Cellular, and Tissue Engineering of the Vascular System* (eds. Fu, B. M. & Wright, N. T.) 219–234 (Springer International Publishing, 2018). 10.1007/978-3-319-96445-4_12 30315548

[10] Dabagh M, Randles A (2019). Role of deformable cancer cells on wall shear stress-associated-VEGF secretion by endothelium in microvasculature. PLoS ONE.

[11] Anvari S, Osei E, Maftoon N (2021). Interactions of platelets with circulating tumor cells contribute to cancer metastasis. Sci. Rep..

[12] Azevedo AS, Follain G, Patthabhiraman S, Harlepp S, Goetz JG (2015). Metastasis of circulating tumor cells: Favorable soil or suitable biomechanics, or both?. Cell Adh. Migr..

[13] Anvari, S., Nambiar, S., Pang, J. & Maftoon, N. Computational Models and Simulations of Cancer Metastasis. *Arch. Comput. Methods Eng.*10.1007/s11831-021-09554-1 (2021).

[14] Shen J, Faruqi AH, Jiang Y, Maftoon N (2021). Mathematical reconstruction of patient-specific vascular networks based on clinical images and global optimization. IEEE Access.

[15] Borg A, Paulsen Husted B, Njå O (2014). The concept of validation of numerical models for consequence analysis. Reliab. Eng. Syst. Saf..

[16] Hou HW (2009). Deformability study of breast cancer cells using microfluidics. Biomed. Microdevices.

[17] Byun S (2013). Characterizing deformability and surface friction of cancer cells. Proc. Natl Acad. Sci. USA.

[18] Chen J (2016). Efficient extravasation of tumor-repopulating cells depends on cell deformability. Sci. Rep..

[19] Wang S, Ye T, Li G, Zhang X, Shi H (2021). Margination and adhesion dynamics of tumor cells in a real microvascular network. PLoS Comput. Biol..

[20] Puleri DF, Randles A (2022). The role of adhesive receptor patterns on cell transport in complex microvessels. Biomech. Model Mechanobiol..

[21] Kienast Y (2010). Real-time imaging reveals the single steps of brain metastasis formation. Nat. Med..

[22] Humayun M (2021). Elucidating cancer-vascular paracrine signaling using a human organotypic breast cancer cell extravasation model. Biomaterials.

[23] Chaw KC, Manimaran M, Tay FEH, Swaminathan S (2006). A quantitative observation and imaging of single tumor cell migration and deformation using a multi-gap microfluidic device representing the blood vessel. Microvasc. Res..

[24] Rosenbluth MJ, Lam WA, Fletcher DA (2008). Analyzing cell mechanics in hematologic diseases with microfluidic biophysical flow cytometry. Lab Chip.

[25] Au SH (2016). Clusters of circulating tumor cells traverse capillary-sized vessels. Proc. Natl Acad. Sci. USA.

[26] Anguiano M (2017). Characterization of three-dimensional cancer cell migration in mixed collagen-Matrigel scaffolds using microfluidics and image analysis. PLoS ONE.

[27] Ren X, Ghassemi P, Babahosseini H, Strobl JS, Agah M (2017). Single-cell mechanical characteristics analyzed by multiconstriction microfluidic channels. ACS Sens..

[28] Nath B (2018). Understanding flow dynamics, viability and metastatic potency of cervical cancer (HeLa) cells through constricted microchannel. Sci. Rep..

[29] Poudineh M (2017). Profiling functional and biochemical phenotypes of circulating tumor cells using a two-dimensional sorting device. Angew. Chem..

[30] Rosendahl P (2018). Real-time fluorescence and deformability cytometry. Nat. Methods.

[31] Van der Meeren L, Verduijn J, Krysko DV, Skirtach AG (2023). High-throughput mechano-cytometry as a method to detect apoptosis, necroptosis, and ferroptosis. Cell Prolif..

[32] Gossett DR (2012). Hydrodynamic stretching of single cells for large population mechanical phenotyping. PNAS.

[33] Urbanska M (2020). A comparison of microfluidic methods for high-throughput cell deformability measurements. Nat. Methods.

[34] Osaki T, Sivathanu V, Kamm RD (2018). Vascularized microfluidic organ-chips for drug screening, disease models and tissue engineering. Curr. Opin. Biotechnol..

[35] Bagnall JS (2015). Deformability of tumor cells versus blood cells. Sci. Rep..

[36] Balogh P, Gounley J, Roychowdhury S, Randles A (2021). A data-driven approach to modeling cancer cell mechanics during microcirculatory transport. Sci. Rep..

[37] Raj A, Sen K (2018). A. Entry and passage behavior of biological cells in a constricted compliant microchannel. RSC Adv..

[38] Raj A, Dixit M, Doble M, Sen AK (2017). A combined experimental and theoretical approach towards mechanophenotyping of biological cells using a constricted microchannel. Lab Chip.

[39] Zhou C, Yue P, Feng JJ (2007). Simulation of neutrophil deformation and transport in capillaries using newtonian and viscoelastic drop models. Ann. Biomed. Eng..

[40] Harvie DJE, Cooper-White JJ, Davidson MR (2008). Deformation of a viscoelastic droplet passing through a microfluidic contraction. J. Non Newton. Fluid Mech..

[41] Leong FY, Li Q, Lim CT, Chiam K-H (2011). Modeling cell entry into a micro-channel. Biomech. Model Mechanobiol..

[42] Shirai A, Masuda S (2013). Numerical simulation of passage of a neutrophil through a rectangular channel with a moderate constriction. PLoS ONE.

[43] Moon JY, Tanner RI, Lee JS (2016). A numerical study on the elastic modulus of volume and area dilation for a deformable cell in a microchannel. Biomicrofluidics.

[44] Xiao LL, Liu Y, Chen S, Fu BM (2016). Numerical simulation of a single cell passing through a narrow slit. Biomech. Model Mechanobiol..

[45] Tan J, Sohrabi S, He R, Liu Y (2018). Numerical simulation of cell squeezing through a micropore by the immersed boundary method. Proc. Inst. Mech. Eng. Part C J. Mech. Eng. Sci..

[46] Zhou L, Feng S, Liu H, Chang J (2019). Dissipative particle dynamics simulation of cell entry into a micro-channel. Eng. Anal. Bound. Elem..

[47] Lim CT, Zhou EH, Quek ST (2006). Mechanical models for living cells—a review. J. Biomech..

[48] Luo YN (2014). A constriction channel based microfluidic system enabling continuous characterization of cellular instantaneous Young’s modulus. Sens. Actuators B Chem..

[49] Reasor DA, Clausen JR, Aidun CK (2012). Coupling the lattice-Boltzmann and spectrin-link methods for the direct numerical simulation of cellular blood flow. Int. J. Numer. Methods Fluids.

[50] Mokbel, M. et al. Numerical simulation of real-time deformability cytometry to extract cell mechanical properties. https://pubs.acs.org/doi/pdf/10.1021/acsbiomaterials.6b00558 (2017). 10.1021/acsbiomaterials.6b00558 33418716

[51] Müller SJ (2021). A hyperelastic model for simulating cells in flow. Biomech. Model Mechanobiol..

[52] Ye T, Phan-Thien N, Lim CT (2016). Particle-based simulations of red blood cells—a review. J. Biomech..

[53] Discher DE, Boal DH, Boey SK (1998). Simulations of the erythrocyte cytoskeleton at large deformation. II. Micropipette aspiration. Biophys. J..

[54] Dao M, Lim CT, Suresh S (2003). Mechanics of the human red blood cell deformed by optical tweezers. J. Mech. Phys. Solids.

[55] Fedosov DA, Caswell B, Karniadakis GE (2010). A multiscale red blood cell model with accurate mechanics, rheology, and dynamics. Biophys. J..

[56] Cimrak, I. & Jancigova, I. *Computational Blood Cell Mechanics: Road Towards Models and Biomedical Applications* (CRC Press, 2018).

[57] Závodszky G, van Rooij B, Azizi V, Hoekstra A (2017). Cellular level in-silico modeling of blood rheology with an improved material model for red blood cells. Front. Physiol..

[58] Ye T, Shi H, Phan-Thien N, Lim CT, Li Y (2018). Relationship between transit time and mechanical properties of a cell through a stenosed microchannel. Soft Matter.

[59] Dao M, Li J, Suresh S (2006). Molecularly based analysis of deformation of spectrin network and human erythrocyte. Mater. Sci. Eng. C..

[60] Pivkin IV, Karniadakis GE (2008). Accurate coarse-grained modeling of red blood cells. Phys. Rev. Lett..

[61] Dupin MM, Halliday I, Care CM, Alboul L, Munn LL (2007). Modeling the flow of dense suspensions of deformable particles in three dimensions. Phys. Rev. E.

[62] MacMECCAN RM, Clausen JR, Neitzel GP, Aidun CK (2009). Simulating deformable particle suspensions using a coupled lattice-Boltzmann and finite-element method. J. Fluid Mech..

[63] Omori T (2011). Comparison between spring network models and continuum constitutive laws: Application to the large deformation of a capsule in shear flow. Phys. Rev. E.

[64] Jančigová I, Kovalčíková K, Bohiniková A, Cimrák I (2020). Spring-network model of red blood cell: from membrane mechanics to validation. Int. J. Numer. Methods Fluids.

[65] Freund A (2003). IL-8 expression and its possible relationship with estrogen-receptor-negative status of breast cancer cells. Oncogene.

[66] Latt, J. et al. Palabos: parallel lattice Boltzmann solver. *Comput. Math. Appl.*10.1016/j.camwa.2020.03.022 (2020).

[67] Bergeaud, V. & Lefebvre, V. SALOME. A software integration platform for multi-physics, pre-processing and visualisation. https://www.osti.gov/etdeweb/biblio/21575789 (2010).

[68] Peskin CS (2002). The immersed boundary method. Acta Numerica.

[69] Poon, C. *Measuring the density and viscosity of culture media for optimized computational fluid dynamics analysis of* in vitro *devices*. http://biorxiv.org/lookup/doi/10.1101/2020.08.25.266221 (2020). 10.1016/j.jmbbm.2021.105024 34911025

